# Thrombocytopenia-absent radius (TAR) syndrome due to compound inheritance for a 1q21.1 microdeletion and a low-frequency noncoding RBM8A SNP: a new familial case

**DOI:** 10.1186/s13039-015-0188-6

**Published:** 2015-11-05

**Authors:** Elisa Tassano, Stefania Gimelli, Maria Teresa Divizia, Margherita Lerone, Carlotta Vaccari, Aldamaria Puliti, Giorgio Gimelli

**Affiliations:** Laboratorio di Citogentica, Istituto Giannina Gaslini, L.go G.Gaslini 5, 16147 Genoa, Italy; Service of Genetic Medicine, University Hospitals of Geneva, 1211 Geneva, Switzerland; U.O.C. Medical Genetics, Istituto Giannina Gaslini, 16147 Genoa, Italy; DiNOGMI, University of Genoa, 16147 Genoa, Italy

**Keywords:** 1q21.1 microdeletion, TAR syndrome, Array-CGH, *RBM8A* SNPs

## Abstract

**Background:**

Thrombocytopenia-absent radius syndrome (TAR; MIM 274000) is a rare autosomal recessive disorder combining specific skeletal abnormalities with a reduced platelet count. TAR syndrome has been associated with the compound inheritance of an interstitial microdeletion in 1q21.1 and a low frequency noncoding *RBM8A* SNP.

**Results:**

Here, we report on a patient with scapulo-humeral hypoplasia, bilateral radio-ulnar agenesis with intact thumbs, bilateral proximal positioning of the first metacarpal, bilateral fifth finger clinodactyly, bilateral radial deviation of the hands, and thrombocytopenia. Molecular studies showed compound heterozygosity for the 1q21.1 microdeletion and the *RBM8A* rs139428292 variant in hemizygous state, inherited from the father and the mother, respectively. A second aborted fetus presented TAR features and 1q21.1 microdeletion.

**Discussion:**

The complex inheritance pattern resulted in reduced expression of Y14, the protein encoded by *RBM8A,* and a component of the core exon-junction complex (EJC) in platelets. Further studies are needed to explain how Y14 insufficiency and subsequent defects of the EJC could cause the skeletal, haematological and additional features of TAR syndrome. In this study, we discuss other factors that could influence the overall phenotype of patients affected by TAR syndrome.

**Conclusion:**

In this study, we discuss other factors that could influence the overall phenotype of patients affected by TAR syndrome.

## Background

Since the introduction of array-CGH analysis, the reported frequency of genomic imbalances associated with specific phenotypes has dramatically increased. Copy number variations (CNV) with incomplete penetrance and variable expressivity have been described in various disorders [1–5]. The association between genomic imbalances and pathological traits can be variable, and additional genetic variations could contribute to the phenotype.

In addition, several other possibilities as epigenetic phenomena, expression or regulatory variation among genes in the vicinity of the unbalanced region, the unmasking of recessive alleles and the possibility of a “two-hit” model, as proposed by Girirajan et al. [6], may account for the phenotypic variability of some genomic diseases.

Human chromosome 1 is rich in segmental duplications, particularly within the pericentromeric region [7–10]. This fact may result in the susceptibility of this region to both pathological and non-pathological CNVs that might have an evolutionary significance.

TAR syndrome (Thrombocytopenia -Absent-Radius) syndrome (MIM 274000) is characterized by thrombocytopenia that may be episodic, congenital skeletal deformities including bilateral absence of radius, shortening and deformity of the ulnae, and occasionally absence of all the long bones in the arm. The fingers and thumbs are always present, while other skeletal anomalies are frequent [11].

A chromosome 1q21.1 microdeletion was identified in 30 patients affected by TAR syndrome [12]. This microdeletion is mediated by Low Copy Repeats (LCRs) that can be at the basis of recurrent DNA rearrangements such as deletions, duplications and inversions through chromosome or chromatid misalignment followed by non-allelic homologous recombination (NAHR) [13–15].

TAR syndrome has a complex pattern of inheritance associated with a minimal common interstitial microdeletion of 200 Kb on chromosome 1q21.1. In several cases, it is inherited from an unaffected parent, while in others it is originated *de novo* and the presence of a 1q21.1 microdeletion is necessary but not sufficient to cause the phenotype.

Recently, it has been shown that compound inheritance of a rare null allele and one of the two low-frequency noncoding SNPs (rs139428292 or rs201779890) in *RBM8A* are crucial for TAR syndrome [16].

Here, we describe the clinical, cytogenetic and molecular features of a 4-month-old boy with TAR syndrome due to co-segregation of 1q21.1 microdeletion and rs139428292. An aborted fetus in the same family presented the same phenotypic features and 1q21.1 microdeletion. We discuss here whether other factors could influence the overall phenotype of TAR syndrome.

### Case report

**Fig. 1:**
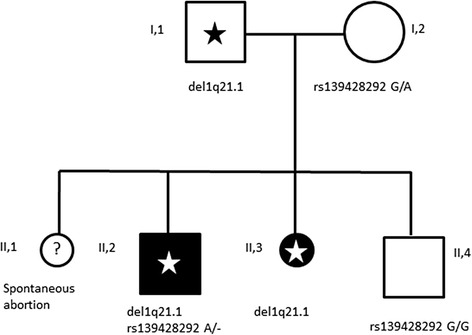
Genealogical tree of the family with TAR syndrome

The family tree is depicted in Fig. 1.

#### Patient 1

**Fig. 2:**
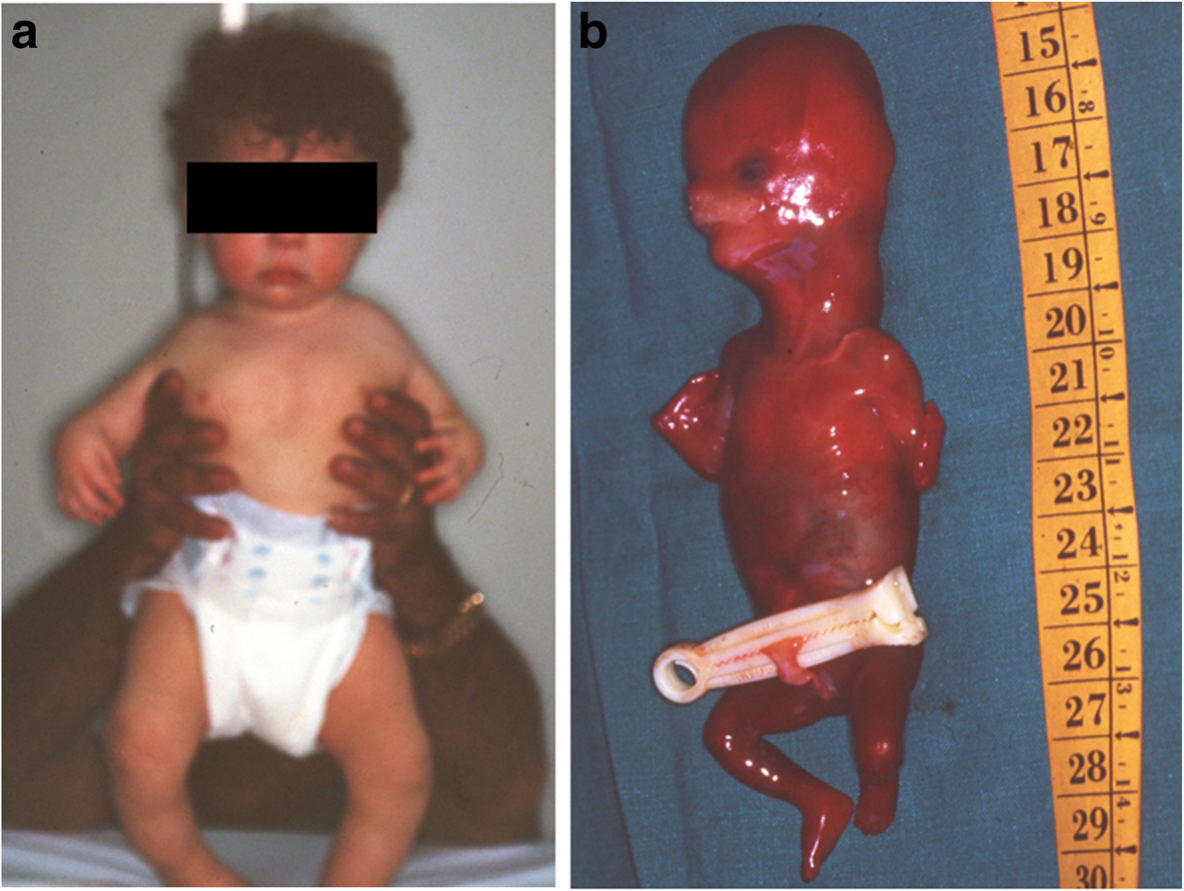
**a** The proband (II-2); bilateral absence of radius with thumb conservation and bilateral *genu varum*. **b** The fetus (II-3); bilateral radial aplasia

The child (II-2) (Fig. 2a) is the first male child of apparently healthy nonconsanguineous parents. The mother and the father were 33 and 45 years old respectively at the time of his birth. Fetal movements were poor. Routine ultrasound examination was normal until 23 weeks of gestation when bilateral radial agenesis was demonstrated. The child was born post-term at 43 weeks of gestation by normal vaginal delivery. Birth weight was 2810 g (10th-25th centile). He was admitted to our institute at 4 months of age. Physical examination showed good nutritional status, forehead and right cheek telangiectasia, scapulo-humeral hypoplasia, bilateral radio-ulnar agenesis with intact thumbs, bilateral proximal positioning of the first metacarpal, bilateral fifth finger clinodactyly and bilateral radial deviation of the hands. X-ray confirmed all these skeletal findings. The child also presented thrombocytopenia (40.000/mmc), PTT (43.7 s). The phenotypic features were characteristic of TAR syndrome (MIM 274000).

#### Patient 2 (Fetus)

During the third pregnancy (II-3) (Fig. 1b), ultrasound examination was performed at 11 weeks of gestation, suggesting the presence of upper limb anomalies. This finding was confirmed at 15th week of gestation. The couple opted for termination of pregnancy. The chromosomal analysis of amniotic cells (400 bands) excluded any visible abnormality. Post-mortem examination of the fetus demonstrated bilateral radial agenesis (Fig. 2b). The mother had a previous pregnancy, which ended in spontaneous abortion at the 2^nd^ month of gestation (II-1). There was no exposure to alcohol, smoking or infections during pregnancy. A subsequent pregnancy resulted in the delivery of a healthy child (II-4).

## Results

**Fig. 3:**
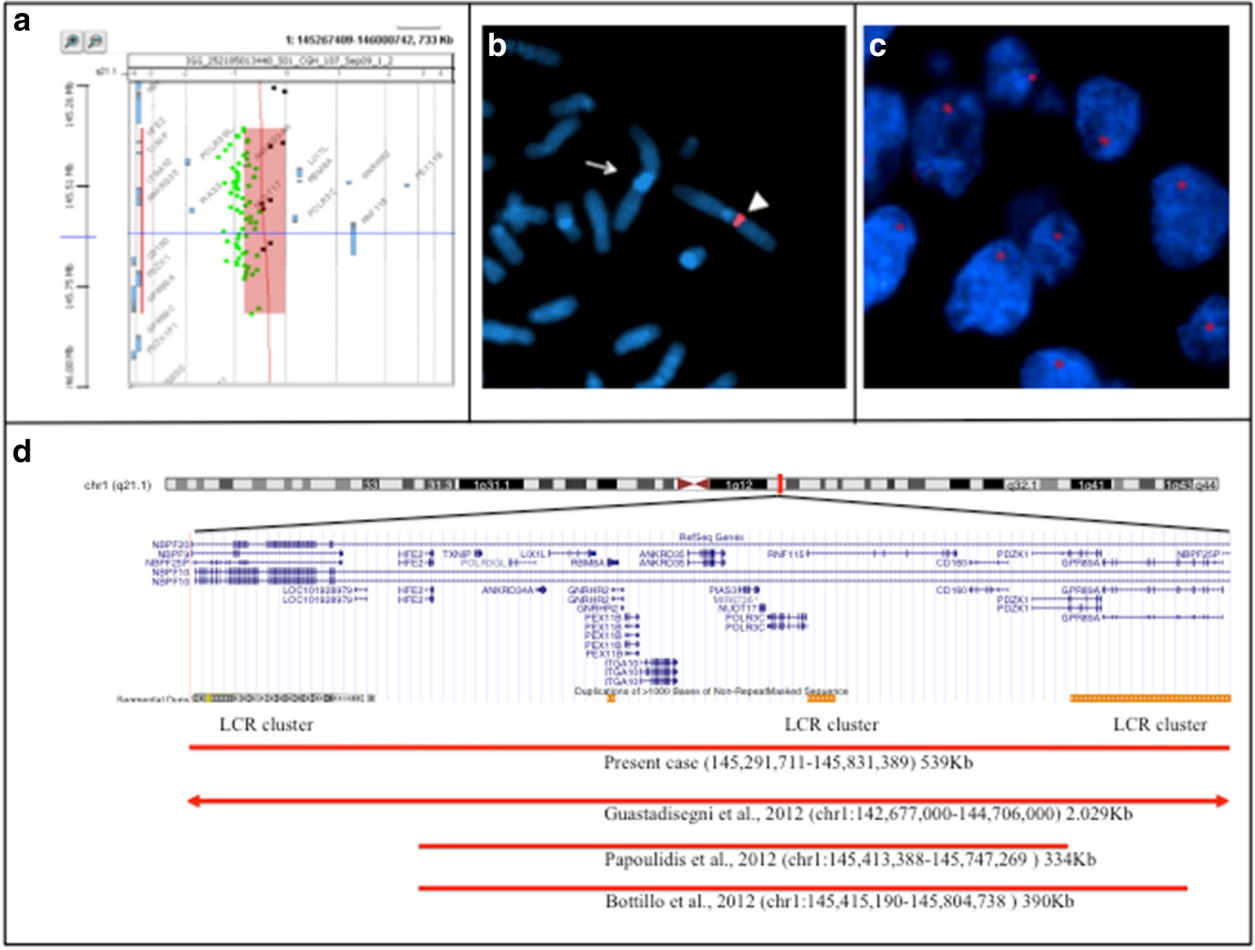
Results of array-CGH and FISH analyses. **a** Array-CGH analysis shows a ~539 Kb microdeletion at 1q21.1 band inherited from the father. **b** FISH confirmation of a hemizygous interstitial 1q21.1 deletion using a BAC probe RP11-105E14 (chr1:145,474,158-145,636,051) (*red*). **c** Interphase FISH with RP11-105E14 (chr1:145,474,158-145,636,051) (*red*) on deparaffinized fetal tissue from patient II, 3. Nuclei show a unique red signal indicating the presence of the deletion. **d** Overview of the 1q21.1 region and its genes and LCRs contents, according to the UCSC Genome Browser (GRCh37/hg19 assembly). The bars indicate the deleted region (*red*) in our patient and the deleted regions in patients reported by Guastadisegni et al. [28], Papoulidis et al. [30] and Bottillo et al. [29].

All patients and their parents showed a normal karyotype. Array-CGH analysis was performed on the available members of the family. An identical 1q21.1 microdeletion (~539 Kb) [arr 1q21.1(145,291,711-145,831,389)x1] was identified in the child with phenotypic features of TAR syndrome (II, 2) as showed in Fig. 3a. The microdeletion was confirmed by FISH (Fig. 3b). The same microdeletion was also present in his apparently normal father (I, 1). An elective abortion was performed because of ultrasound findings of upper limb anomalies, strongly suggestive of TAR syndrome (II, 3). FISH analysis by BAC RP11-105E14 (chr1:145,474,158-145,636,051) on archived specimens confirmed the presence of the “TAR microdeletion” (Fig. 3c). In the same family, one spontaneous abortion (II, 1) was reported but unfortunately not investigated, as biological specimens were not available. The deleted region contains 12 MIM genes: *NBPF20* (MIM 614007; neuroblastoma breakpoint family, member 20), *NBPF10* (MIM 614000; neuroblastoma breakpoint family, member 10), NBPF9 (MIM 613999; neuroblastoma breakpoint family, member 9), *HFE2* (MIM 602390; hemochromatosis type 2A), *TXNIP* (MIM 606599; thioredoxin-interacting protein), *RBM8A* (MIM 605313; RNA-binding motif protein 8A), *GNRHR2* (MIM 612875; gonadotropin-releasing hormone receptor 2), *PEX11B* (MIM 603867; peroxisome biogenesis factor 11B), *ITGA10* (MIM 604042; integrin, alpha-10), *PIAS3* (MIM 605987; protein inhibitor of activated STAT3), *CD160* (MIM 604463; CD160 antigen), *PDZK1* (MIM 603831; PDZ domain-containing 1). It is interesting to note the presence of the *LIX1L* (Gene ID: 128077; Lix1 homolog (chicken) like) gene (Fig. 3d).

**Fig. 4:**
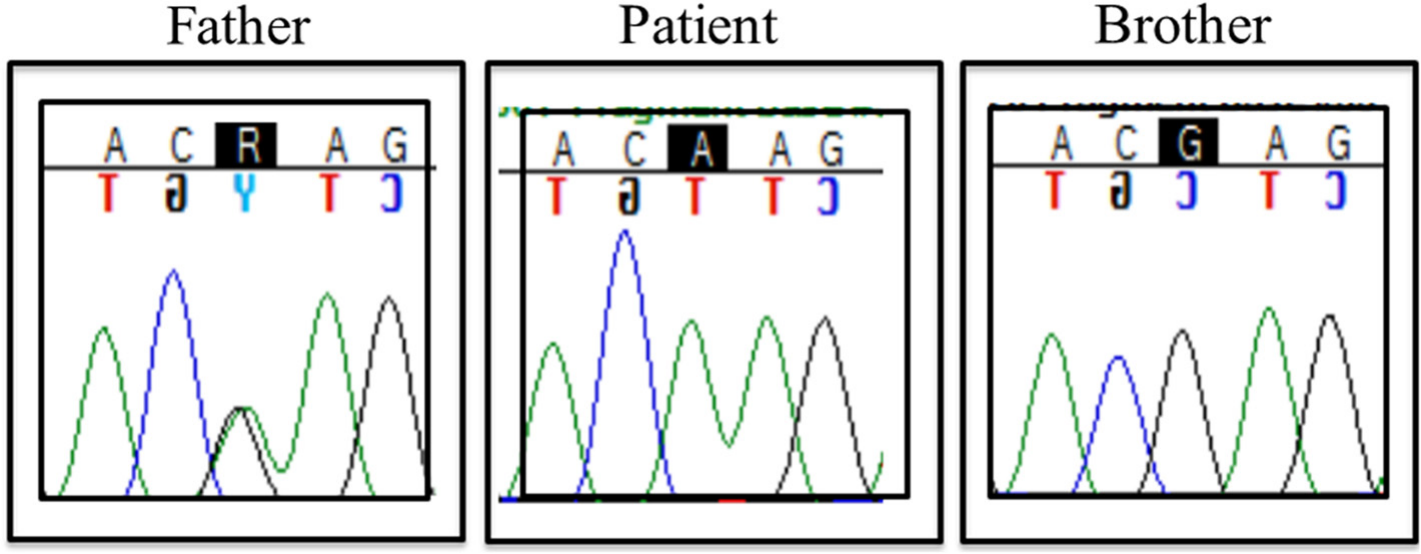
Results of Sanger sequencing of the rs139428292 variant (G > A) obtained from the patient and his unaffected mother and his unaffected brother

The family were analysed for the rs139428292 (G > A) and the rs201779890 (G > C) SNPs of *RBM8A* gene by direct sequencing. The analysis of rs139428292 showed that the patient harboured the minor (A) allele, which was inherited from his healthy mother. The father and the healthy brother were both homozygous for the major (G) allele. All family members carried the major (G) allele of rs201779890 in a homozygous state (Fig. 4).

## Discussion

We describe an identical 1q21.1 microdeletion in affected and non-affected members of a family with TAR syndrome. The same rearrangement was firstly described in 30 patients affected by Thrombocytopenia-Absent-Radius (TAR) syndrome (MIM 274000), a rare malformation syndrome characterized by hypo-megakaryocytic thrombocytopenia and bilateral absence of the radius in the presence of both thumbs [12]. The microdeletion was inherited from either the unaffected mother or the unaffected father in 75 % of cases. Recently, it has been proposed to consider this syndrome as a complex trait disease requiring at least two genetic changes: the rare microdeletion and another, relatively frequent, genetic variation that acts as a modifier of the other one. The family described in this study was referred to our centre for TAR syndrome and was investigated by array-CGH. A 1q21.1 microdeletion of ~539 Kb overlapping the TAR critical region, inherited from the phenotypically normal father, was identified in the two affected cases (II, 2 and II, 3). The identification of the interstitial microdeletion in affected offspring and the paternal inheritance are in agreement with the findings of Klopocki and collaborators [12]. The presence of 1q21.1 microdeletion is necessary but not sufficient to cause the phenotype, since, in the majority of cases, TAR traits can develop only in the presence of a second modifier as the low-frequency regulatory SNP reported by Albers et al. [16].

In fact, a study identified two rare single nucleotide polymorphisms (SNPs) in the regulatory region of the *RBM8A* gene that are involved in TAR syndrome through the reduction of the expression of the RBM8A-encoded Y14 protein [16]. The first allele (rs139428292 G > A), which is located in the 5′ untranslated region (UTR) of the gene, was demonstrated to have a minor allele frequency (MAF) of 3.05 %, and the second allele (rs201779890 G > C), located in the first intron of the gene, exhibited a MAF of 0.42 %, in 7504 healthy individuals from Cambridge BioResource (Cambridge, UK) [16, 17]. Our patient had inherited a low-frequency 5′UTR SNP (rs139428292 G > A) from his mother and the 1q21.1 microdeletion from his father.

A number of patients with TAR syndrome were reported, but in almost all these cases the precise coordinates of the deleted regions were not available and in others no mutation analysis of *RBM8A* SNP had been performed [18–27].

At our knowledge, in only three TAR cases with 1q21.1 deletion, analysed by array-CGH, the precise coordinates of the deletion has been reported [28–30] (Fig. 3d). Two are prenatal cases and one postnatal. All have the classical TAR features (thrombocytopenia, upper limbs and hands anomalies); two have inherited the low-frequency 5′ UTR SNP (rs139428292 G > A) and the minimal overlapping 1q21.1 deletion region ranging from 145,415,190 to 145,747,269 [29, 30].

Particularly, the child affected by TAR syndrome associated with Langerhans cell histiocytosis described by Guastadisegni et al. [28] showed a larger deletion (2.029 Kb) and a significant downregulation of the commonly deleted genes. The mainly implicated gene in the syndrome is *RBM8A*, a gene encoding the exon-junction complex subunit member Y14. Y14 is a small protein with an RNA-binding domain and one of the four components of EJC, which is involved in basic cellular functions such as nuclear export and subcellular localization of specific transcripts, translational enhancement, nonsense-mediated RNA decay and splicing [31]. It has a crucial role during embryonic developmental [32]. The level of Y14 was found to be significantly lower in the platelets of TAR patients. It is noteworthy, that Y14 may regulate the expression of genes involved in the proliferation of hematopoietic cells. However, it is not clear how a deficiency in Y14 exerts its effects at a cellular level and in particular how it affects the production of megakaryocytes and platelets. A possible explanation for this observation could be that deficiency in EJC could have an influence on the defective cell signalling in megakaryocyte. Similarly, no relation has been found between *RBM8A* deletion and TAR skeletal anomalies. To provide an explanation for the skeletal abnormalities observed in TAR syndrome, Albers et al., [24] assumed that, in addition to a tissue-dependent effect, it is possible that the regulatory SNPs had developmental stage–dependent consequences keeping as an example the Mecom gene encoding Evi1 that is expressed in a transient manner in emerging limb buds in mouse [33].

Albers et al., [24] explained that TAR phenotype could be also influenced from other factors such as environmental factors altering gene expression, incomplete penetrance or additional modifier alleles. We speculate that other genes in the 1q21.1 region other than *RBM8A* could influence the phenotype of TAR syndrome. It is interesting to note that, among the several genes with a known function located within the region, the *PIAS3* gene could be indicated as the most conspicuous candidate for thrombocytopenia and the *Lix1L* gene, known be transiently expressed during chick hind-limb development, could be proposed as the candidate gene for limb abnormalities [34–36].

## Conclusions

In conclusion, we reported on a new familial case of TAR syndrome in a child and in a fetus carrying a 1q21.1 microdeletion and the low-frequency 5′UTR SNP (rs139428292 G > A). We also focused on two genes (*PIAS3* and *Lix1L*), contained in the deleted region, which could play a role in determining some important aspects of the phenotype of this syndrome. Obviously, further in-depth studies are needed to clarify the possible role of these genes.

## Methods

### Cytogenetics, fluorescent in situ hybridization and array-CGH analyses

Karyotypes were performed on peripheral blood of patients and their parents. Fluorescent in situ hybridization (FISH) analysis was performed following the manufacturer’s instructions (Vysis, Abbott Molecular, Illinois, U.S.A.). BAC clone was selected from the human library RPCI-11 according to the UCSC Human Genome Assembly (GRCh37/hg19). Array-CGH using the Human CGH Kit 244 K (Agilent Technologies, Palo Alto, CA, U.S.A.) covering the whole genome with a 8.9 Kb overall median probe spacing was performed following the manufacturer’s protocol.

### FISH analysis on paraffin-embedded fetal tissues

To prepare paraffin-embedded tissue sections fixed on positively charged slides we cut 4–5 mμ thick paraffin sections using a microtome. Floating sections were mounted on positively charged slides. Slides were treated by Paraffin Pretreatment Kit (Abbott Molecular Inc., IL, USA) to deparaffinise specimens. Then, the slides were treated with protease and hybridization with BAC probes was performed according to the appropriate Vysis protocol.

### Genotyping of 5′ UTR and intronic SNP of the *RBM8A* gene

The genotypes of the rs139428292 and the rs201779890 SNPs were obtained. Genomic DNA was isolated from peripheral blood leukocytes using a standard protocol. The genomic regions encompassing the two SNPs were PCR amplified, purified and then sequenced on both strands using the BigDye dideoxy-terminator chemistry on an ABI 3100 DNA sequencer (Applied Biosystems, Foster City, CA). Primers used for both amplification and sequencing were the following: rs139428292 (Fw: CCTTTCCCCTCTGCGACA; Rv: CCCAGCCTCGTGAAGATCTA) and rs201779890 (Fw: TAGATCTTCACGAGGCTGGG; Rv: GGGGCGGAATCTCTAATCCA).

### Ethics Statement

The current study was performed using peripheral blood of the members of the family treated at the Istituto Giannina Gaslini, Genova, Italy. The parents of the patient gave written informed consent allowing molecular and genetic studies. We didn’t request approval by Review Board of our institution, because our study request only classical and molecular cytogenetic analyses. For cytogenetics analyses are sufficient only written informed consent of the parents (DM 21 dicembre 2007). The informed consents of the parents were previously authorized by the Review Board of our institution. We didn’t conduct research outside our country of residence. We didn’t approach the local authorities before beginning work on this study. The full name of the ethics committee of our institution is Comitato di Etica per la Ricerca Scientifica Biomedica, per la Buona Pratica Clinica e per la Sperimentazione dei Farmaci.

### Consent

Written informed consent was obtained from the patient's parents for the publication of this report and any accompanying images.
